# POSTURAL BALANCE AND FUNCTIONAL MUSCLE STRENGTH IN THE HANDS AND LEGS ONE YEAR AFTER HOSPITALISATION DUE TO COVID-19

**DOI:** 10.2340/jrm.v57.42763

**Published:** 2025-06-15

**Authors:** Lena RAFSTEN, Alexandra LARSSON, Annie PALSTAM, Hanna PERSSON

**Affiliations:** 1 Institute of Neuroscience and Physiology, Department of Clinical Neuroscience and Rehabilitation Medicine, Sahlgrenska Academy, University of Gothenburg, Gothenburg; 2 Department of Occupational Therapy and Physiotherapy, Sahlgrenska University Hospital, Gothenburg; 3 School of Health and Welfare, Dalarna University, Falun, Sweden

**Keywords:** COVID-19, postural balance, muscle strength, hand, leg

## Abstract

**Objective:**

The aim of the study was to investigate postural balance and functional muscle strength over 1 year following hospital discharge due to COVID-19 and identify possible differences depending on age, sex, and level of hospital care.

**Design:**

A prospective longitudinal study.

**Subjects:**

A total of 164 participants were included.

**Methods:**

Postural balance, functional leg strength, and functional hand strength were evaluated. Change over time and differences between groups were investigated.

**Results:**

At the 1-year follow-up postural balance was improved (*p* = 0.001), as well as strength in the hands (*p* = 0.001), and legs (*p* = 0.001). Participants treated at an intensive care unit (ICU) had impaired functional muscle strength in the hands but not in the legs 1 year after discharge. Functional muscle strength in dominant hand on discharge, age, and previous level of physical activity were associated with having more impaired functional muscle strength in the dominant hand 1 year after discharge.

**Conclusion:**

Functional muscle strength and postural balance after COVID-19 improved significantly from discharge to the 1-year follow-up although nearly half of the patients still had impaired functional muscle strength 1 year after COVID-19 hospitalization.

*Trial registration:* FoU i Sverige (Research & Development in Sweden, Registration number: 274476, registered 2020-05-28).

## LAY ABSTRACT

This study investigate the presence of sequelae after COVID-19 infection. Studies have reported that at least 70% of patients in the post-acute stage have persisting symptoms 1 year after COVID-19. Persistence of symptoms can be anxiety, depression, sleep disturbances, impaired postural balance, and impaired muscle strength. To help people that suffer from those symptoms we aimed to investigate postural balance and functional muscle strength over 1 year following hospital discharge due to COVID-19 infection to identify possible rehabilitation needs. In all, 164 participants concluded the 1-year follow up. On discharge, just over half of the participants had impaired postural balance compared with about 13% at the 1-year follow-up. On discharge, 65% had impaired functional muscle strength in the hands compared with 41% at the 1-year follow-up. On discharge, 94.5% had impaired functional muscle strength in the legs compared with 53% 1 year after hospital discharge. We concluded that although functional muscle strength and postural balance improved significantly from hospital discharge to 1-year follow-up, nearly half of the patients still had impaired functional muscle strength after 1 year. Older age meant poorer recovery of functional muscle strength and postural balance after COVID-19.

Severe COVID 19 requires hospitalization, and in worst cases treatment in the intensive care unit (ICU) (1). In Sweden, approximately 7% of those with confirmed COVID-19 infection required hospital care, and of those, approximately 9–11% needed intensive care during the first and second waves (2) prior to the vaccination programme. Today, billions of doses of COVID-19 vaccine have been administered worldwide (3), and the number of new COVID-19 infections tends to fluctuate seasonally with an increase in cases during the winter months (4). Though most people with COVID-19 recover from the acute infection within weeks of illness, some individuals continue to experience the presence of sequelae (5, 6). Several studies have reported persisting symptoms 1 year after COVID-19 (7–9), with one study indicating that at least 70% of patients in the post-acute stage experience ongoing symptoms (10). A systematic review has shown that survivors of critical COVID-19 have experienced residual symptoms, such as fatigue, reduced functional status, and reduced quality of life (11). Other studies have reported a combination of symptoms such as breathlessness, neuropsychological symptoms, joint or muscle pain, headache, and dizziness when standing (12, 13). These post-COVID conditions are commonly known as “long COVID” (14), which is defined as a wide range of new, returning, or ongoing health problems experienced 12 or more weeks after initial infection.

Some retrospective studies have presented clinical aspects of severe COVID-19 and concluded that symptom persistence is a primary determinant of mental health outcome, such as anxiety, depression, sleep disturbances, and post-traumatic stress symptoms (15). It has also been reported that greater hand strength on admission predicts a shorter length of hospital stay for patients with mild to moderate COVID-19 (16), and a greater loss of muscle mass is associated with a higher frequency of post-acute sequelae of COVID-19 (17).

Postural balance refers to the ability to maintain body stability and proper function in stationary positions or while moving, such as sitting, standing, or walking (18). Impaired postural balance and muscle strength are potential consequences after hospitalization for COVID-19, but their long-term impacts have not been deeply investigated, and the long-term consequences remain unknown. However, it has been shown that postural sway is increased in individuals recently recovered from COVID-19 (19), and an association between lower-limb muscle strength and static and dynamic balance control has been demonstrated (20). By examining and following these aspects during the first year after severe COVID-19, we can identify potential rehabilitation needs related to postural balance and muscle strength.

The aim of the present study was to investigate postural balance and functional muscle strength over 1 year following hospital discharge due to COVID-19 infection, and to identify possible differences depending on age, sex, and level of hospital care.

## Materials and methods

This study is a part of a longitudinal study, the “Life in the time of COVID” study in Gothenburg (GOT-LOCO). This trial is registered in FoU i Sverige (Research & Development in Sweden, Registration number: 274476, registered 2020-05-28). Patients treated for COVID-19 were consecutively included from 5 hospitals in the Västra Götaland region (VGR) in Sweden from July 2020 to February 2021, during the first and second wave of the COVID-19 pandemic. The 1-year follow-up was conducted from July 2021 to March 2022. This study was designed according to the Strengthening the Reporting of Observational Studies in Epidemiology (STROBE) guidelines for observational studies (21).

### Study population

The inclusion in GOT-LOCO was conducted at the hospital, with the following inclusion criteria: hospitalized due to COVID-19, non-contagious at enrolment, age ≥ 18 years, expected hospital stay ≥ 5 days, and previously lived in their own housing. Patients were excluded if they were unable to provide informed consent, with comorbidities with a high 1-year mortality risk (i.e., palliative care or metastatic cancer), or were not Swedish residents.

Patients fulfilling the inclusion criteria were identified by the study coordinator or a local test leader (a physical or occupational therapist working at each hospital) and invited to participate. Written informed consent was obtained from all participants before data collection.

### Data collection

Data collection was performed by physiotherapists and occupational therapists at the hospital before discharge and 1 year after hospital discharge. Standardized tests used in this study were based on functionality considering the current pandemic restrictions. Demographic data such as age, level of hospital care, and length of hospital stay were collected from medical charts. The 1-year follow-up was conducted as an outpatient visit at the hospital or in the participant’s home if necessary. To ensure standardized data collection, a study coordinator trained the test leaders, and the physiotherapists and occupational therapists involved in the study. All participants were assessed following a standardized test protocol.

### Postural balance

Dynamic postural balance was assessed using the 10 Metre Walk Test (10MWT) at both comfortable gait speed (CGS) and fast gait speed (FGS). The time and number of steps were recorded (22). The 10MWT has demonstrated excellent reliability (23, 24). Static postural balance was assessed using the single-leg stance test, with time recorded (25, 26). Participants were allowed to use walking aids but not receive physical assistance (supervision was allowed). Time started when the participant began walking (standing start with toes behind the start mark) and ended when the participant crossed the finish line. The participant must cross the finish line with their whole body for the timer to stop. Before starting, participants were informed orally about the test. Instructions included “Now you should walk at your chosen gait speed” or “Now you should walk as fast as you can, safely”. Cheering was not allowed during the test. A value of >12 s on the 10MWT comfortable speed (27) and >10 s on the single-leg stance test (28, 29) were considered indicative of impaired postural balance

### Functional muscle strength in the hands

Functional muscle strength in the hands was assessed using the JAMAR hand dynamometer test (30–32), which has excellent interrater reliability (33, 34). The hand dynamometer test measures isometric hand strength. It was performed 3 times in each hand and the mean of the 3 tests was calculated, along with the percentage of normative values (30).

### Functional muscle strength in the legs

Functional muscle strength in the legs was assessed using the 30-second chair stand test (30 s CST) (35, 36), which has excellent interrater reliability (35). In the 30 s CST, the number of correctly performed chair stands within 30 s was recorded (37). The test was performed using a chair without armrests, and the participants crossed their arms over their chest during the test. They were instructed to stand up straight each time and were allowed 1 practice trial before the test. If the participant could not perform the chair stand test without using their hands for support, a score of 0 was recorded. Timing began with the cue “now” and then the number of chair stands was counted for 30 s. The instruction was: “Do as many chair stands as possible, as fast as you can, and safely. Start when I say ‘now’”. If the participant could not perform the test as instructed, this was counted as 0 chair stands. We aspired to have a standardized seat height of 45 cm, but in the acute setting patients were isolated, and an available chair was then used. At the acute assessment, the chair seat height varied between 40 and 54 cm, with a mean of 45 cm. At the follow-up the seat height was 45 cm in all performed tests.

### Covariates

Walking ability was assessed using the functional ambulating category (FAC) (38, 39), a 6-point assessment of ambulating ability that determines how much human support is required for walking, regardless of the use of assistive device. Scores range from 0 (non-functional ambulation) to 5 (can walk freely on any surface) (39). The participants’ physical activity levels before the COVID-19 and 1 year after discharge were assessed using the Saltin–Grimby Physical Activity Level Scale (SGPALS) (40, 41), which rates physical activity level during leisure time ranging from 1 (physically inactive) to 4 (regular hard physical training for competitive sports) (40, 41). The Post-COVID Functional Status (PCFS) scale was used to evaluate the impact on functional status post-acute COVID-19 infection, both before and 1 year after hospital discharge. The PFCS is a self-assessed questionnaire that summarizes the patient’s view of impairment after COVID-19 infection (42). Scores range from 0 (not feeling affected at all) to 4 (being totally physically impaired).

### Statistical analysis

Data are presented as a percentage, mean±SD, or medians and interquartile ranges (IQR) for the total group, and based on age and level of care for subgroups. The group of younger participants corresponds to those < 65 years, and the older group corresponds to those ≥ 65 years. Groups were also defined by level of care (whether participants had received ICU treatment or not). For group differences in descriptive data, the χ^2^ test and Mann–Whitney *U* test were used. Change over time was analysed with the Wilcoxon signed-rank test.

Logistic regression analysis was performed to identify potential predictors of impaired postural balance or impaired functional muscle strength in the hands and legs 1 year after hospital discharge. The 10MWT CGS scores were dichotomized into “impaired postural balance” or “not impaired postural balance” (27). Strength in the dominant hand was assessed using a hand dynamometer and was dichotomized to “impaired functional muscle strength” (< 100% of the mean normative values based on age and sex), and “not impaired functional muscle strength” (≥ 100% of the mean normative values based on age and sex) (30). The 30-s CSTs were dichotomized into “impaired functional muscle strength in the legs” (< normal value for sex and age), and “not impaired functional muscle strength in the leg” (≥ normal value for sex and age) (43). The cut-off value for 60-year-olds was used for participants < 60 years, with values of < 14 for men and < 12 for women. Ten possible independent variables were identified: age, sex, length of hospital stay, level of hospital care, SGPALS, FAC, PCFS, 10MWT, functional muscle strength in the dominant hand, and 30-s CST on discharge from hospital. Variables were tested for multicollinearity using Spearman’s rank correlation coefficient test (*r*_s_) and variables with r_s_ < 0.7 were included in the models. Because the level of hospital care and number of hospital days were correlated ≥ 0.7, only the number of hospital days was included in the models, leaving 9 independent variables applicable for the multivariable regression analysis. The independent variables were dichotomized as follows: FAC 0 = dependent (level 0–3) and 1 = independent (level 4–5), SGPALS 0 = physically inactive (level 1) and 1 = physically active (level 2–4), PCFS 0 = functional limitations (level 2–4) and 1 = no functional limitations (level 0–1). In the analyses, “zero” is always the reference. To test the goodness of fit and accuracy of the model, the Hosmer and Lemeshow test (*p* > 0.05, good fit) and Nagelkerke R^2^ test (a value closer to 1 was anticipated) were used. The area under the receiver operating characteristic curve (ROC, a value closer to 1 was anticipated) was used to evaluate the model’s ability to discriminate participants with impaired postural balance and impaired functional muscle strength at the one-year follow-up.

## Results

**Fig 1:**
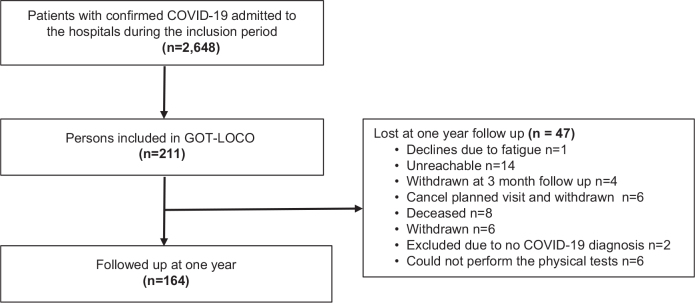
Flowchart of the study participants.

In total, 211 participants were included in the GOT-LOCO study. Of these, 164 were examined at the 1-year follow-up (Fig. 1). Some 32% were women, and the median age was 65 years (IQR 56–74) (Table I); 52% had been treated in the ICU and 49% had received continued rehabilitation after discharge.

**Table I:** Descriptive characteristics stratified by sex and subgroups

Item	Total group (164)	*p*-value	Male (*n* = 111)	Female (*n* = 53)	*p*-value	Year > 65 (*n* = 86)	*p*-value	Year < 65 (*n* = 78)	*p*-value	ICU admitted (*n* = 85)	*p*-value	Non ICU (*n* = 79)	*p*-value
Age, years, mean (SD)	64.2 (12.98)		64.5 (11.2)	63.6 (16.3)	0.47	74.1 (6.7)		53.4 (9.1)		62.4 (11.9)		66.1 (13.9)	**0.047**
Median (IQR)	65.0 (56–74)		65 (57–74)	65 (53.5–76.5)		74(69–78)		56 (47–60)		64 (55–71)		68 (59–76)	
Sex, males (%)	111 (67.7)					59 (68.6)		52 (66.7)	0.88	64 (75.3)		47 (59.5)	**0.015**
Hospital stay, days, mean (SD)	34.3 (37.1)		38.9 (41.8)	24.4 (21.5)	0.06	35.4 (31.7)		33.04 (42.32)	0.06	51.8 (43.3)		15.3 (12.6)	**< 0.001**
Median (IQR)	19 (10–43.7)		20 (11–54)	17 (9–31)		22.5 (11.7–45.8)		15.5 (9–34.3)		38 (20–70)		11 (8–17)	
Smoking, Yes/no/former/unclear	5/44/40/71		1/33/31/44	4/11/9/27		2/16/29/37		3/28/11/34		3/29/17/36		2/15/23/39	
Pulmonary condition before COVID-19, Yes/no/missing	43/100/18		22/73/12	21/27/6		24/55/7		19/43/16		24/50/11		19/50/10	
SGPALS, median (IQR)													
** **Acute (*n*=162)	2 (1–2)	0.238				2 (2)	0.78	2 (1–3)	0.059	2 (1–2)	0.573	2 (1–2)	**0.017**
12 months (*n*=164)	2 (1–2)		2(1–2)	2(1–2)	**0.04**	2 (4)		2 (1–2)		2 (2–2)		2 (1–2)	
BI, median (IQR)													
** **Acute (*n*=158)	90 (75–100)	**< 0.001**				85 (60–100)	**< 0.001**	100 (80–100)	**< 0.001**	80 (60–90)	**< 0.001**	100 (90–100)	**< 0.001**
12 months, (*n*=99)	100 (100–100)		100(100–100)	100(100–100)		100 (100–100)		100 (100–100)		100 (100–100)		100 (100–100)	
FAC, median (IQR)													
Acute (*n*=160)	4 (3–5)	**< 0.001**				4 (3–4.5)	**< 0.001**	5 (4–5)	**< 0.001**	4 (3–4)	**< 0.001**	5 (4–5)	**< 0.001**
12 months (*n*=164)	5 (5–5)		5(5–5)	5(4–5)	**0.01**	5 (4–5)		5 (5–5)		5 (5–5)		5 (4–5)	
PCFS, median (IQR)													
Acute (*n*=162)	3 (2–4)	**< 0.001**				3 (2–4)	**< 0.001**	3 (1–3)	**< 0.001**	3 (2–4)	**< 0.001**	3 (1–3)	**< 0.001**
12 months (*n*=163)	2 (0–3)		1(0–2)	2(1–3)	**0.002**	2 (0–3)		1.5 (0–2)		2 (0–3)		1 (0–3)	

SD: standard deviation; IQR: interquartile range; ICU: intensive care unit; SGPALS: Saltin–Grimby Physical Activity Scale; BI: Barthel Index; FAC: Functional Ambulatory Classification; PCFS: Post COVID-19 Functional Status. Significant *p* values are marked in bold.

### Postural balance

**Fig. 2:**
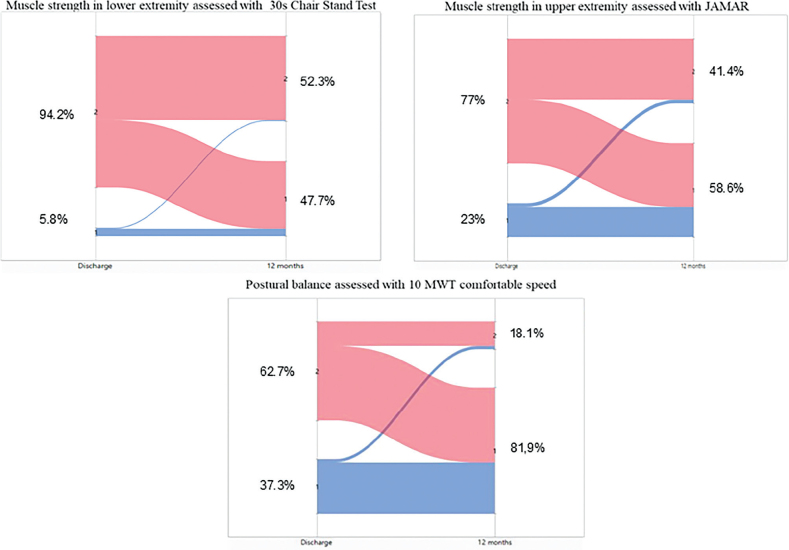
Sankey diagram showing change over time dichotomized as normal functional muscle strength/postural balance or impaired functional muscle strength/postural balance. 1 = normal functional muscle strength/postural balance, 2 = impaired functional muscle strength/postural balance, 10MWT = 10 Metre Walk Test.

A total of 115 participants were able to perform the single-leg stance test for at least 10 s at the 1-year follow-up. Younger participants were able to perform the test for a longer duration with older participants (*p* < 0.001), but no difference was found based on the level of hospital care (*p* = 0.591). An improvement in walking speed and the number of steps was observed from discharge to the 1-year follow-up (Table II). On discharge, 51.8% of the participants had impaired postural balance according to the cut-off in the 10MWT CGS, compared with 13.5% at the 1-year follow-up (*p* < 0.001) (Fig. 2). Younger participants walked significantly faster (comfortable and fast speeds) (*p* = 0.001) and took fewer steps (*p* = 0.01) compared with older participants, both on discharge and at the 1-year follow-up (Table II). After 1 year, no statistical difference in postural balance was found based on level of hospital care (Table II).

**Table II:** Postural balance and functional strength: change over time and subgroup analysis

Item	All participants (*n* = 164)			Subgroup analysis at 12 months					
	Before discharge	1-year after discharge	*p-*value	Age < 65 (*n* = 78)	Age ≥ 65 (*n* = 86)	*p-*value	ICU (*n* = 85)	No ICU (*n* = 79)	*p-*value
Postural balance*, CGS, seconds, mean (SD)	16.4 (7.8) (*n*=140)	9.7(3.1) (*n*=151)	**< 0.001**	8.8 (1.9)	10.7 (3.7)	**< 0.001**	9.9 (3.3)	9.5 (2.8)	0.58
Postural balance*, CGS, steps, mean (SD)	21.9 (6.5)	16.5 (3.7)	**< 0.001**	15.5 (2.7)	17.4 (4.4)	**0.01**	16.9 (4.1)	15.9 (3.3)	0.07
Postural balance**, FGS seconds, mean (SD)	11.1 (5.5) (*n*=133)	6.8 (1.9) (*n*=147)	**< 0.001**	6.3 (1.5)	7.4 (2.2)	**< 0.001**	6.7 (1.4)	7.1 (2.4)	0.73
Postural balance**, FGS, steps, mean (SD)	18.1 (5.8)	13.8 (2.8)	**< 0.001**	13.2 (2.7)	14.5 (2.8)	**0.01**	13.8 (2.6)	13.8 (3.1)	0.42
Functional leg strength* mean (SD)	5.9 (3.7) (*n*=159)	11.6 (5.5) (*n*=159)	**< 0.001**	13.3 (5.2)	10.2 (5.3)	**< 0.001**	11.5 (5.7)	13.2 (4.7)	0.58
Functional hand strength* right hand, kilos, mean	27.1(14.4) (*n*=156)	36.6 (13.7) (*n*=162)	**< 0.001**	40.7 (14.8)	32.8 (11.5)	**< 0.001**	35.1 (15.4)	37.9 (11.8)	**0.001**
left hand kilos, mean	25.6 (13.4) (*n*=156)	35.1 (14.2) (*n*=162)	**< 0.001**	38.9 (15.4)	31.3 (11.9)	**0.001**	35.6 (12.9)	34.3 (15.4)	**< 0.001**

* Postural balance measured with 10MWT CGS.

** Postural balance measured with 10MWT FGS.

*Functional leg strength measured with 30 CST. *Functional hand strength measured with JAMAR.

CGS: comfortable gait speed; FGS: fast gait speed; ICU: intensive care unit; 10MWT: 10-metre walking test; 30 CST: 30 second chair stand test; SD: standard deviation. Significant *p* values are marked in bold.

### Associations between independent variables and postural balance

When checked for collinearity, 9 independent variables met the criteria to be included to the model. In the multivariable regression, none of the independent variables contributed significantly to the model (Table III).

**Table III:** Logistic regression predicting likelihood of having impaired postural balance 1 year after hospital discharge

Predictors of postural balance assessed with 10MWT, CGS (*n* = 125)	OR	95% CI	*p*-value
Sex (ref. male)	2.57	0.81–8.19	0.12
Age (years older)	1.04	0.99–1.09	0.09
Hospital stay (days longer)	1.01	0.99–1.02	0.49
Functional status (PCFS, ref. functional limitations )	0.76	0.17–3.33	0.72
Physical activity level (SGPALS, ref. inactive)	0.72	0.24–2.18	0.77
Walking ability (FAC, ref. dependent)	0.42	0.12–1.55	0.19
Dynamic postural balance (10MWT CGS discharge.seconds)	1.05	0.98–1.13	0.14

Hosmer and Lemeshow test: *p* = 0.551, Nagelkerke R squared = 0.247, AUC = 0.239.

AUC: area under the curve, FAC: Functional Ambulation Category, CGS: comfortable gait speed, PCFS: Post COVID-19 Functional Status, ref.: Reference, SGPALS: Saltin–Grimby Physical Activity Level Scale, 10MWT: 10 Metre Walk Test.

### Functional muscle strength in the hands

Functional muscle strength improved in both the dominant and non-dominant hands from discharge to 1 year after hospital discharge. On discharge, 65% of participants had impaired functional muscle strength in both hands, compared with 41% at the 1-year follow-up (see Fig. 2). Younger participants were significantly stronger than older participants (see Table II), with a mean value of 40.7 kg (14.8), compared with 32.8 kg (11.5) at the 1-year follow-up. Participants who had been treated in the ICU had significantly lower functional muscle strength in both hands at 1- year follow-up, with a mean value of 35.1 kg (15.4) compared with 37.9 kg (11.8) in those who were not treated in the ICU (see Table II).

### Associations between independent variables and functional muscle strength in the hands

When checked for collinearity, 9 independent variables met the criteria to be included in the model. In the multivariable regression model, the strongest predictor of impaired functional muscle strength in the dominant hand was functional muscle strength in dominant hand on discharge from the hospital (OR = 0.94, 95% CI 0.91–0.97) (Table IV), along with older age (OR = 0.95, 95% CI 0.92–0.98). Furthermore, prior level of physical activity (OR = 0.38, 95% CI 0.17–0.84) made a significant contribution to the model, where lower physical activity before falling ill with COVID-19 was associated with more impaired functional muscle strength in the dominant hand 1 year after discharge (Table IV).

**Table IV:** Logistic regression predicting likelihood of having impaired functional muscle strength in dominant hand 1 year after hospital discharge

Predictors of impaired muscle strength in dominant hand assessed with JAMAR (*n* = 150)	OR	95% CI	*p*-value
Sex (ref. male)	0.78	0.31–2.02	0.61
Age (years, older)	0.95	0.92–0.98	**0.002**
Hospital stay (days longer)	0.99	0.98–1.01	0.38
Post-COVID functional status (PCFS, ref. functional limitations)	0.73	0.28–1.84	0.49
Physical activity level (SGPALS, ref. inactive)	0.38	0.17–0.84	**0.017**
Walking ability (FAC, ref. dependent)	1.05	0.36–3.06	0.93
Functional hand strength (JAMAR dominant hand at discharge, ref. lower)	0.94	0.91–0.97	**0.002**

Hosmer and Lemeshow test: *p* = 0.537, Nagelkerke R squared = 0.247, AUC = 0.761.

AUC: area under the curve, FAC: Functional Ambulation Category, PCFS: Post Covid Functional Scale, ref.: Reference, SGPALS: Saltin–Grimby Physical Activity Level Scale. Significant *p* values are marked in bold.

### Functional muscle strength in the legs

Ninety participants were able to perform the 30-s CST according to instructions on discharge, compared with 141 at the 1-year follow-up (see Table II). On discharge, the mean number of chair stands was 5.9 (3.7). On average, the men performed 6.2 (3.9) and the women 5.5 (3.2) chair stands. At the 1-year follow-up the average increased significantly (*p* < 0.001) to 11.6 (5.5) chair stands. The men performed 12.1 (5.6) and the women 10.7 (5.2) (Table II). On discharge, 94.5% had impaired functional muscle strength in the legs compared with 53% 1 year after hospital discharge (see Fig. 2). Older participants had significantly lower functional leg strength than younger participants at the 1-year follow-up; however, functional leg strength did not differ based on the level of hospital care (Table II).

### Associations between independent variables and functional leg strength

In the logistic regression, 9 independent variables met the criteria to be included in the model. In the multivariable regression model, the strongest predictor for impaired leg muscle strength 1 year after discharge was the 30-s CST on discharge from the hospital (OR = 0.86, 95% CI 0.77–0.95) (Table V).

**Table V:** Logistic regression predicting likelihood of having impaired functional muscle strength in legs 1 year after hospital discharge

Predictors of impaired functional muscle strength in legs assessed with 30 s chair stand (*n* = 150)	OR	95% CI	*p*-value
Sex (ref. male)	0.66	0.31–1.41	0.28
Age (years, older)	0.99	0.97–1.03	0.72
Hospital stay (days, longer)	1.01	0.99–1.02	0.78
Functional scale (PCFS, ref. functional limitations)	0.95	0.40–2.27	0.91
Physical activity level (SGPALS, ref. inactive)	0.52	0.23–1.16	0.11
Walking ability (FAC, ref. dependent)	1.30	0.47–3.61	0.62
Functional leg strength (30 sec CST at discharge, number of chair stands)	0.86	0.77–0.95	**0.003**

Hosmer and Lemeshow test, *p* = 0.450. Nagelkerke R squared = 0.174, ACU = 0.705.

AUC: area under the curve, CST: chair stand test, FAC: Functional Ambulation Category, PCFS: Post Covid Functional Scale, ref: Reference scale, SGPALS: Saltin–Grimby Physical Activity Level Scale. Significant *p* values are marked in bold.

## DISCUSSION

This longitudinal study demonstrated that most of the participants hospitalized due to COVID-19 had impaired postural balance and functional muscle strength at the time of hospital discharge. A year later, nearly half of the patients still had impaired functional muscle strength, and 13% had impaired postural balance. We found that functional muscle strength in both the hands and legs was lower in older participants compared with younger ones 1 year after discharge. Additionally, younger participants had better postural balance compared with older participants at the 1-year follow-up. The study also revealed that participants treated in the ICU had lower functional hand strength at the 1-year follow-up compared with those who were not treated in the ICU. However, the level of hospital care did not predict either decreased functional muscle strength in the arms and legs or impaired postural balance 1 year after discharge from the hospital.

In this study, only 60% and 47% had normal strength in the hands and the legs, respectively, at the 1-year follow-up. Previous studies have shown negative impacts on muscle strength 3-4 months post-hospital discharge due to COVID-19 (44, 45). However, the participants’ muscle strength prior to COVID-19 infection was unknown, which limits our ability to fully understand the extent of the decline in strength.

In the present study, participants treated in the ICU were significantly weaker in the hands but not in the legs 1 year after discharge, compared with participants who were not treated in the ICU. This aligns with studies reporting ICU admission to be associated with decreased muscle strength, compared with age- and sex-matched normative values, 6 months to 1 year after discharge (46, 47).

Strength in the dominant hand on hospital discharge was one of the most important factors for functional hand strength 1 year later. Furthermore, age was significantly associated with hand strength 1 year after discharge, which is expected as muscle fibre loss begins around the age of 50 (48, 49). In addition, prior physical activity levels had a significant association with functional hand strength at the 1-year follow-up. This could be reasonable, as participants who were more physically active and fit prior to the illness could be expected to continue that behaviour after discharge.

Functional strength in the legs on discharge was the most important factor of functional leg strength at the 1-year follow-up, although age did not significantly affect leg strength. Findings from the same cohort, 3 months after discharge, showed that nearly 25% of the participants reported an inability to participate in heavy maintenance tasks and activities involving both arms and hands (50). If this limitation persisted for a year, it could explain differences in functional hand and leg strength observed at the 1-year follow-up.

Older participants had worse postural balance compared with younger ones, which is also plausible to anticipate and consistent with other studies (51). This is in line with studies that have mapped postural balance in the general population, showing that increased age is related to impaired postural balance (52). Neither sex nor the level of hospital care, as a proxy for disease severity, affected postural balance 1 year after discharge. Additionally, postural balance on discharge was not associated with the outcome 1 year later.

A strength of the present study is the structured and standardized data collection by trained physiotherapists and occupational therapists, both on discharge and during follow-up, either at the hospital or in the participants’ homes. This flexible approach allowed 81% of participants in the cohort to be assessed. A second strength is that the assessments used in this study have excellent inter-rater reliability (23, 24, 33, 34). Additionally, we had a regional cohort whereby participants from 5 hospitals in the region were included, which allowed for a large cohort of participants from both rural and urban areas. While we aimed to include participants consecutively, the risk of selection bias cannot be ruled out. Several factors may have contributed to missed inclusion such as clinicians being too busy due to a heavy workload, patients being too fatigued to participate, unknown information as to whether patients were contagious or not, as well as many patients being transferred between different wards, hospitals, and in the region. It is a weakness that we do not have any data on how many patients were missed for inclusion due to these factors. Choosing to dichotomize outcomes into “impaired” and “not impaired” could also be seen as weakness, as participants near the cut-off values may have been classified based on arbitrary assumptions. Furthermore, when dichotomizing leg strength we had only normative values for people 60 years and older, which led to us also using this as the cut-off for participants < 60 years. Additionally, previous status or other symptoms that may have affected postural balance or motor skills post-COVID were not taken into account, which is another limitation. This study did not include any control group, although the data were compared with normative values for age- and sex-matched individuals, which could serve as a proxy for a control group.

In conclusion, although functional muscle strength and postural balance improved significantly from hospital discharge to the 1-year follow-up, nearly half of the participants still had impaired functional muscle strength after 1 year. Older age meant poorer recovery of functional muscle strength and postural balance after COVID-19.
